# Effects of Sleep Deprivation on Phase Synchronization as Assessed by Wavelet Phase Coherence Analysis of Prefrontal Tissue Oxyhemoglobin Signals

**DOI:** 10.1371/journal.pone.0169279

**Published:** 2017-01-03

**Authors:** Lingguo Bu, Ming Zhang, Jianfeng Li, Fangyi Li, Heshan Liu, Zengyong Li

**Affiliations:** 1 Key Laboratory of High Efficiency and Clean Mechanical Manufacture, School of Mechanical Engineering, Shandong University, Jinan, P.R. China; 2 Interdisciplinary Division of Biomedical Engineering, Faculty of Engineering, The Hong Kong Polytechnic University, Kowloon, Hong Kong, SAR P.R. China; 3 Key Laboratory of Rehabilitation Aids Technology and System of the Ministry of Civil Affairs, National Research Center for Rehabilitation Technical Aids, Beijing, P.R. China; Centro de Neurociencias de Cuba, CUBA

## Abstract

**Purpose:**

To reveal the physiological mechanism of the decline in cognitive function after sleep deprivation, a within-subject study was performed to assess sleep deprivation effects on phase synchronization, as revealed by wavelet phase coherence (WPCO) analysis of prefrontal tissue oxyhemoglobin signals.

**Materials and Methods:**

Twenty subjects (10 male and 10 female, 25.5 ± 3.5 years old) were recruited to participate in two tests: one without sleep deprivation (group A) and the other with 24 h of sleep deprivation (group B). Before the test, each subject underwent a subjective evaluation using visual analog scales. A cognitive task was performed by judging three random numbers. Continuous recordings of the near-infrared spectroscopy (NIRS) signals were obtained from both the left and right prefrontal lobes during rest, task, and post-task periods. The WPCO of cerebral Delta [HbO_2_] signals were analyzed for these three periods for both groups A and B.

**Results:**

Six frequency intervals were defined: I: 0.6–2 Hz (cardiac activity), II: 0.145–0.6 Hz (respiratory activity), III: 0.052–0.145 Hz (myogenic activity), IV: 0.021–0.052 Hz (neurogenic activity), V: 0.0095–0.021 Hz (nitric oxide related endothelial activity) and VI: 0.005–0.0095 Hz (non-nitric oxide related endothelial activity). WPCO in intervals III (F = 5.955, p = 0.02) and V (F = 4.7, p = 0.037) was significantly lower in group B than in group A at rest. During the task period, WPCO in intervals III (F = 5.175, p = 0.029) and IV (F = 4.585, p = 0.039) was significantly lower in group B compared with group A. In the post-task recovery period, the WPCO in interval III (F = 6.125, p = 0.02) was significantly lower in group B compared with group A. Reaction time was significantly prolonged, and the accuracy rate and F_1_ score both declined after sleep deprivation.

**Conclusions:**

The decline in WPCO after sleep deprivation indicates reduced phase synchronization between left and right prefrontal oxyhemoglobin oscillations, which may contribute to the diminished cognitive function.

## Introduction

Sleep is a neural state, during which both memory consolidation and homeostatic preservations take place [1, 2]. During sleep, significant brain activity in different regions is occurring to form networks for optimal information processing in a waken state [3–5]. Sleep deprivation has various effects on human performance and neural functions, which are reflected in different description levels [6]. It could reduce a subject’s vigilance, raise the risk in decision making, and affect cognitive behavior, as well as a decrease sustained attention, cause an increased error rate and memory impairment [7]. The neuronal effects of sleep deprivation can be predicted based on activation during a normal rested condition [8]. Study on effects of sleep deprivation on brain functional networks reveals differential sensitivity of brain areas to sleep deprivation [9]. Changes in cerebral activation are shown to occur as a function of sleep deprivation, and these changes are correlated with cognitive performance [10]. Hence, the research into brain function mechanisms is very important when studying of the effects of sleep deprivation on cognitive function.

The prefrontal cortex (PFC) plays an important role in cognitive function [11]. The PFC is used to perform advanced neural information processing functions, including judgment, analysis, thinking, and operation [12]. Moreover, the PFC is vulnerable to sleep deprivation, which influences various cognitive functions [13]. The dynamic changes of blood oxygen while memory tasks are being performed suggest that the PFC is important in the working memory of the human neural network and performing control operations [14]. The alteration of short-time perception is associated with left PFC activation [13], and right PFC activity reflects the ability of the subject to overcome sleepiness while performing memory tasks [15].

Near-infrared spectroscopy (NIRS) can identify the volume of regional cerebral blood by detecting the changes of Delta [HbO_2_] and deoxygenated hemoglobin (Delta [dHb]) concentrations [16]. NIRS has been used to study the brain activities during active tasks and rest periods [17]. This technology has some advantages in the study of brain function [18, 19], such as portability, convenience, low cost, and imposes less constraints on test subjects. Also, this technology is suitable for observing brain functions while performing cognitive tasks.

Wavelet phase coherence (WPCO) can be used to discover possible correlations between two signals by assessing the match between two instantaneous phases from the signals [20]. The phase synchronization between the left and right prefrontal oscillations is considered to be related to the cognitive functions [21, 22]. However, little information is known about the effects of sleep deprivation on the phase synchronization. In this study, sleep deprivation was hypothesized to affect the phase synchronization between the left and right prefrontal oscillations in various characteristic frequency ranges. The objective of this study was to assess the effects of sleep deprivation on phase synchronization of prefrontal tissue oxyhemoglobin signals using the WPCO method. The results of this study would provide a new insight into the mechanism of cognitive functions changes after sleep deprivation.

## Materials and Methods

### Subjects

To compute the minimum sample size, the required power level, pre-specified significance level and the population effect size need to be considered [23]. In this study, the minimum sample size of 10 can achieve required statistical index (α>0.95, β<0.2). So we recruited 20 subjects to get higher power level. 20 healthy subjects (10 male and 10 female, 25.5 ± 3.5 years) were recruited from Shandong University. Among them, one subject was excluded due to the abnormal sleep quality index. Thus, the data used were 19.

The subjects all met the following criteria: (1) right-handed; (2) no history of neurological or psychiatric diseases; and (3) no current drug treatments. The subjects’ sleep quality was evaluated for a month through Pittsburgh sleep quality index questionnaire [24]. This questionnaire consists of 9 self-rating, 5 rating by others items, and 18 other items which are combined to form 7 factors. Each factor was scored by 0–3 levels, and the total score was calculated from the sum of all the factors. A higher total score means worse sleep quality. A good sleep quality refers to that the subjects have habitual good sleeping habits including sleeping no less than 6.5 h each night for the past month, sleeping no later than 1:00 am and waking no later than 9:00 am [5]. Scores of less than 6 indicate good sleep quality [24, 25]. The average sleep quality of the subjects in the current study was 4.1, with a range from 2 to 6. Stimulants, alcohol, tea, and coffee were not allowed within the 24 hours before the test. The experimental procedures were approved by the Human Ethics Committee of Shandong University and were in accordance with the ethical standards specified by the Helsinki Declaration of 1975 (revised in 1983). The participants provided their written informed consent and the ethics committees approved this consent procedure.

### Procedures

Subject information, including height, weight, gender, systolic and diastolic blood pressures, and duration of sleep from the previous night, were recorded before the test, as shown in Table 1. Blood pressure was measured before both of with and without deprivation. Each subject was to participate in two tests: one test without sleep deprivation (group A) and the other test with 24 h sleep deprivation (group B). Sleep deprivation was carried out in a specific room from 7:00 am of the first day to 7:00 am of the next day. The time interval between the two tests was one week. For participants who took the deprivation session prior to the rested wakefulness session, the possibility of residual effects of sleep deprivation on cognition needed to be minimized [5]. During sleep deprivation test, subjects were allowed to engage in leisure activities, such as watching TV, surfing the internet, playing cards, or reading books. However, they were forbidden from participating in strenuous activities. The test started at 7:30 am.

**Table 1:** Basic information of the experimental subjects.

Characteristic	Basic information
Age (years)	25.5 ± 3.5
Height (cm)	167.8 ± 7.2
Weight (kg)	58.7 ± 11.2
Female sex	50%
Body mass index (kg/m^2^)	20.6 ± 4.5
Group A_Systolic blood pressure (mmHg)	116.8 ± 11.5
Group A_Diastolic blood pressure (mmHg)	73.1 ± 5.2
Group B_Systolic blood pressure (mmHg)	117.4 ± 15.7
Group B_Systolic blood pressure (mmHg)	74.1 ± 12.9
Duration of sleep from the previous night (h)	7.4 ± 0.5

All the subjects were made aware of the purpose, process and important matters regarding the test prior to commencement. To avoid the interference of external factors, the tests were performed in a special room. Before the test, the subjects were instructed to adjust their breathing and relax while sitting for 10 min [26] and stabilizing their emotions to ensure the accuracy of experimental data collected. They took the subjective scale test first and then the subjects completed a self-rated psychological measurement twice using 100 mm Visual Analog Scales (VAS) [27]. Participants were asked to label the line with their current subjective evaluation and were evaluated on six aspects of alertness, flexibility, comfort, excitement, pain and fatigue.

**Fig 1:**
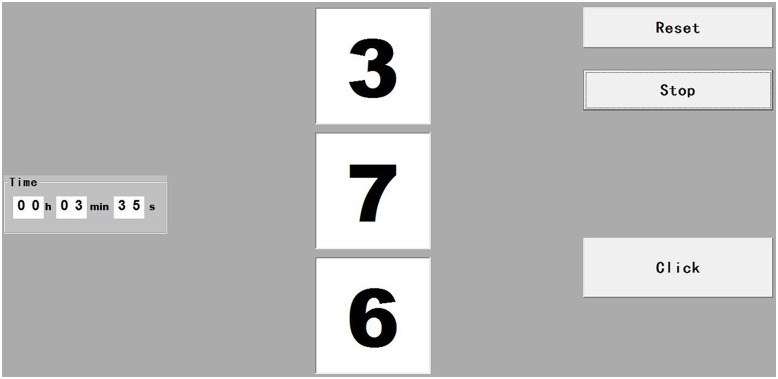
The interface of the cognitive task when operating.

The test, either without or after deprivation, was divided into three parts: rest, task, and detection of post-task recovery as follows: During the first part of the test, each subject took a 20-min rest period, during which they kept their eyes closed, relaxed, refrained from moving, and avoided any structured mental activity [28]. During the 20-min rest period, the lab staff quietly observed the behavior of the subjects (such as the presence of nodding off and other sleep-like behaviors). In this study, the frequency range measured was 0.005 Hz—2 Hz. In order to achieve reliable detection of coherence with the frequency 0.005 Hz, a minimum measurement time of 1000 s (16 min) was required [29]. Therefore, a sampling time of 20 min was used. Cerebral oxygenation signals in the test were detected and recorded using an oximeter. After the 20-min rest period, there was a pause, and the task period started within 10 s. All subjects performed the vigilance task for 20 min. Fig 1 shows the interface used for the vigilance task in which a group of three random numbers (from 0 to 9) was displayed on the screen and changed randomly every 1.3 s. The subjects were to immediately click on a button when the three numbers shown were different odd numbers. The click time was recorded. Data were discarded if accuracy was below 80%, indicating that participants did not maintain a high attention level. The subjects were instructed to familiarize themselves with protocol for performing the task prior to the experiment. They were also instructed to avoid sudden movements during the task. Meanwhile, cerebral oxygenation signals were detected and recorded using a NIRS tissue oximeter. Finally, the 20 subjects maintained their relaxed state after the vigilance task and their recovery state was detected continuously by the oximeter for 20 min. During the post-task recovery period, the subjects also had to keep their eyes closed, stay relaxed, refrain from any movements, and avoid any structured mental activity.

### Measurement

A four-channel NIRS tissue oxygenation monitor (TSAH-200, Tsinghua University, China), was used to collect [HbO_2_] of brain tissues of the subjects. This NIRS system was used and verified in our previous studies [21, 22]. During signal collection, the near-infrared detector was placed on the left and right prefrontal lobes, simultaneously collecting data and analyzing hemodynamics in the biological tissues. Oxygenated and reduced hemoglobin reflected the oxygen content in the tissues. Particularly, Delta [HbO_2_] reflected the oxygen consumption rate. The total hemoglobin level was used to measure the degree to which the tissue was filled with blood ensuring a continuous oxygen supply to the brain tissue.

The NIRS consisted of two light sources with LED light-emitting diodes, operating at emission wavelengths of 760 nm and 850 nm respectively and matching PIN diodes which were used as detectors. The probes were placed at left and right prefrontal lobe which is 1.5 cm from the horizontal middle of the brain and located 2 cm above the brow to be kept away from the longitudinal sinuses and frontal sinuses [30].

The prefrontal area of each subject was cleaned with medical alcohol. To ensure that the NIRS device was not interfered with by other lights during the test, the detector was connected to the subjects using a flexible adhesive tape and a rubber band and the probe of the detector was wound around the subjects' head with a piece of bandage. The sampling frequency of NIRS equipment was 10 Hz. The subjects were instructed to limit sudden changes in their body posture to avoid experimental error caused by human motion.

### Data pre-processing

The data pre-processing method was described in our previous studies [21, 22] and the collected signals were expected to have outliers and trend terms. Signal pre-processing was used to remove these outliers and trend terms so that the overall signal facilitated the later analysis of coherence. A moving average method and a Butterworth filter were chosen in this study to achieve this.

The calculation of a moving average is a method used to improve signal-noise ratio by using an averaging method, and is widely used in signal processing [31]. The principle is to calculate the average value of 2N+1 data points around each data point, and then use the average value to replace each of the original data points. In general, 2N+1 points before the data point or N points before and after the data point can be used. In this paper, 5 points before the abnormal point was taken to replace an abnormal point, and the expression is [31, 32]:

$$y(n)=\frac{1}{2N+1}\sum_{i=1}^{2N+1}x(n-i)$$ (1)

In this expression, x(n) expressed the original time series, y(n) indicated the time series after moving average, and N = 2. The abnormal point was selected by setting the amplitude threshold value to 5, and points above this threshold were considered to be abnormal.

### Wavelet-based coherence analysis

This method was described in our previous studies [21, 22]. Briefly, the WPCO method uses the phase information of the signal to evaluate the correlation of two signals. It identifies possible connectivity through quantitatively representing the degree to which the instantaneous phase of two signals in the time-series continuous process is kept consistent [20]. The calculation method of significant WPCO values was described in our previous study [33]. In this study, wavelet-based coherence method was used to assess phase synchronization. WPCO is particularly valuable when it comes to low-frequency components, which significantly contribute to cardiovascular status [34].

The WPCO value was calculated using the frequency domain amplitude of the instantaneous phase difference, which was averaged over time [35]. Firstly, two time series, *x*_1_(*t*_*n*_) and *x*_*2*_(*t*_*n*_), were obtained from the wavelet transform and their corresponding instantaneous phases were *ϕ*_1_(*f*, *t*_*n*_) and *ϕ*_2_(*f*, *t*_*n*_) respectively. Secondly, the instantaneous phase difference between the two signals was calculated.

$$\Delta \phi (f,t_{n})=\phi_{1}(f,t_{n})-\phi_{2}(f,t_{n})$$ (2)

Thirdly, cosΔ*ϕ*(*f*, *t*_*n*_) and sinΔ*ϕ*(*f*, *t*_*n*_) were averaged in the time domain. Finally, the phase coherence function was defined as [35]:

$$WPCO(f)=\sqrt{{\langle \text{cos}\Delta \varphi (f)\rangle }^{2}+{\langle \text{sin}\Delta \varphi (f)\rangle }^{2}}$$ (3)

**Fig 2:**
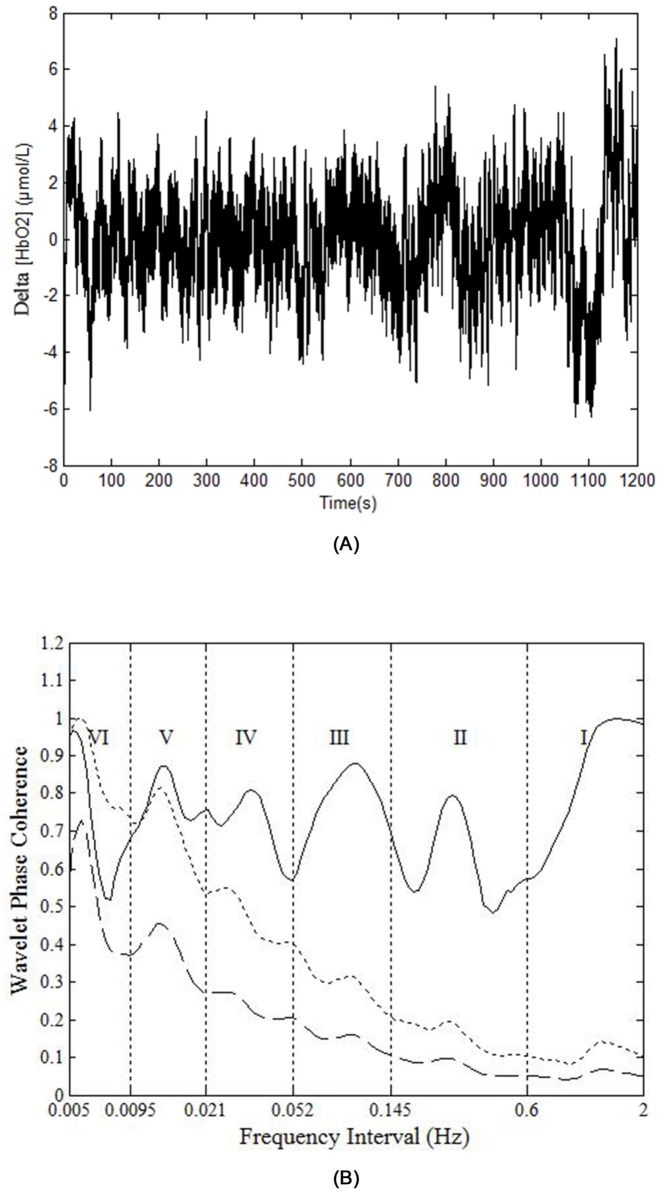
An example of the phase coherence of the left and right prefrontal Delta [HbO_2_] oscillations, and the mean and two standard deviations of AAFT surrogate signals. (A) Raw time series of a Delta [HbO_2_] signal. (B) The solid line shows the wavelet phase coherence of two Delta [HbO_2_] signals. The dashed line and the dotted line show the mean and two standard deviations above the mean for the coherence calculated from 100 AAFT surrogate signals, respectively. In (B), the vertical lines indicate the outer limits of the frequency intervals: I (0.6–2 Hz), II (0.145–0.6 Hz), III (0.052–0.145 Hz), IV (0.021–0.052 Hz), V (0.0095–0.021 Hz), and VI (0.005–0.0095 Hz).

**Fig 3:**
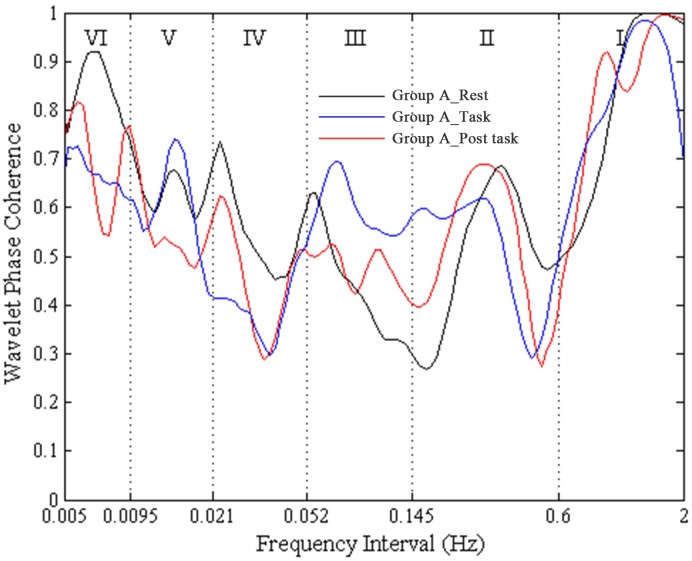
An example of plot of WPCO of the Delta [HbO_2_] signals measured from the left and right prefrontal lobes in group A from a subject during rest, task, and post-task periods. The black line shows the WPCO of two Delta [HbO_2_] signals during the rest period in group A. The red line shows the WPCO of two Delta [HbO_2_] signals during the task period in group A. The blue line shows the WPCO of two Delta [HbO_2_] signals during the task period in group A.

**Fig 4:**
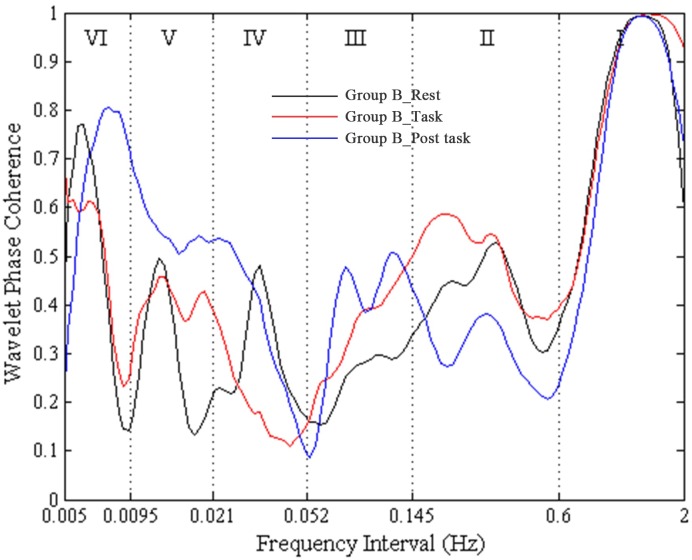
An example of plot of WPCO of the Delta [HbO_2_] signals measured from the left and right prefrontal lobes in group B from a subject during rest, task, and post-task periods. The black line shows the WPCO of two Delta [HbO_2_] signals during the rest period in group B. The red line shows the WPCO of two Delta [HbO_2_] signals during the task period in group B. The blue line shows the WPCO of two Delta [HbO_2_] signals during the task period in group B.

A high WPCO value indicates high agreement between the phase angles of Delta [HbO_2_] signals of the left and right prefrontal lobes, which further signifies the synchronization of neural activations of the said lobes. Fig 2 shows an example of the phase coherence of the left and right prefrontal Delta [HbO_2_] oscillations, and the mean and two standard deviations of amplitude-adjusted Fourier transform (AAFT) surrogate signals. Fig 3 shows an example of plot of WPCO of the delta [HbO_2_] signals measured from the left and right prefrontal lobes in group A from the same subject during rest, cognitive task, and post-task periods. Fig 4 shows an example of plot of WPCO of the delta [HbO_2_] signals measured from the left and right prefrontal lobes in group B from the same subject during the rest, cognitive task, and post-task periods.

### Statistical analysis

All values were expressed as means and standard deviations. The data of the subjects were tested for normality (Kolmogorov–Smirnov test) and homogeneity of variance (Levene test) to ensure they meet the assumptions required by parameter analysis. Two-way repeated measures ANOVA were used to analyze the influence of sleep deprivation and task status (rest, task, and post-task recovery), as well as the relationship between them. One-way ANOVA was used to compare the difference between groups A and B among the rest, task, and post-task recovery periods, respectively. A p<0.05 difference was considered to be statistically significant.

Then, a one-way ANOVA was used to compare the rest, task, and post-task recovery of group A with those of group B and then the post-hoc test was performed. Each test was performed using Bonferroni comparison tests.

## Results

### Wavelet phase coherence

**Fig 5:**
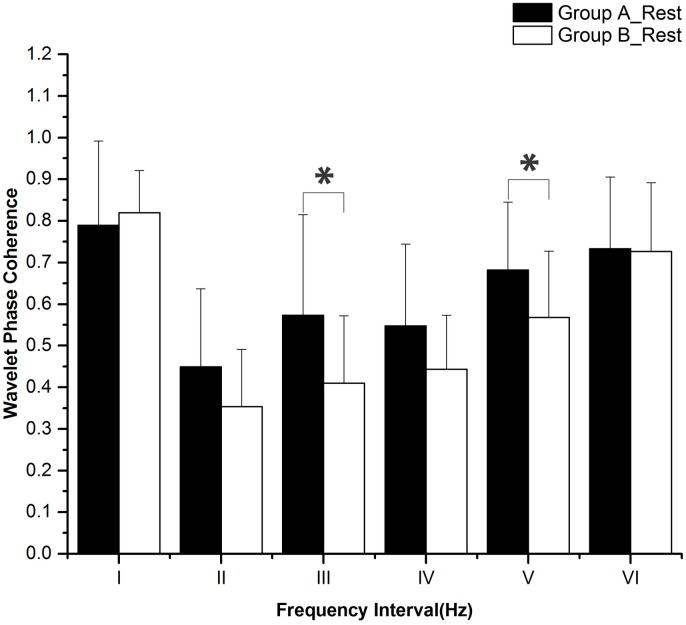
WPCO comparison in six frequency intervals at rest between groups A and B. Significant differences are marked with *p<0.05 between the two groups. Frequency intervals: I (0.6–2 Hz); II (0.145–0.6 Hz); III (0.052–0.145 Hz); IV (0.021–0.052 Hz); V (0.0095–0.021 Hz); and VI (0.005–0.0095 Hz).

**Fig 6:**
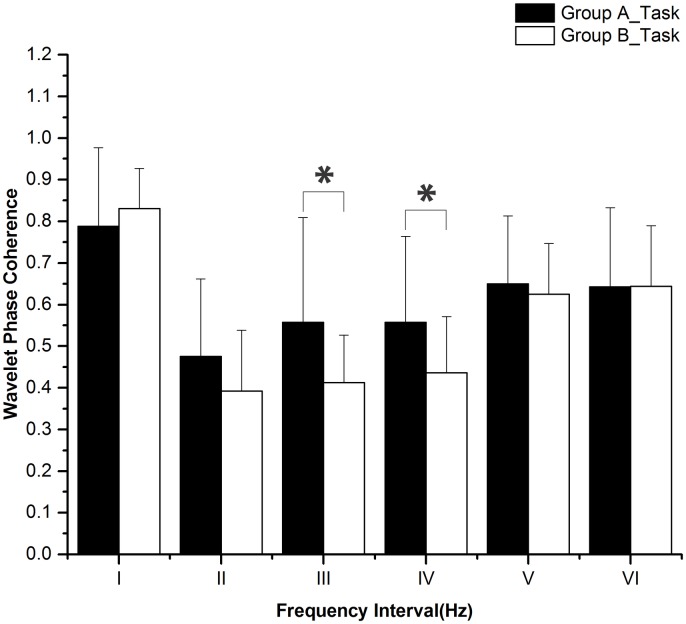
WPCO comparison in six frequency intervals in task between groups A and B. Significant differences are marked with *p<0.05 between the two groups. Frequency intervals: I (0.6–2 Hz); II (0.145–0.6 Hz); III (0.052–0.145 Hz); IV (0.021–0.052 Hz); V (0.0095–0.021 Hz); and VI (0.005–0.0095 Hz).

**Fig 7:**
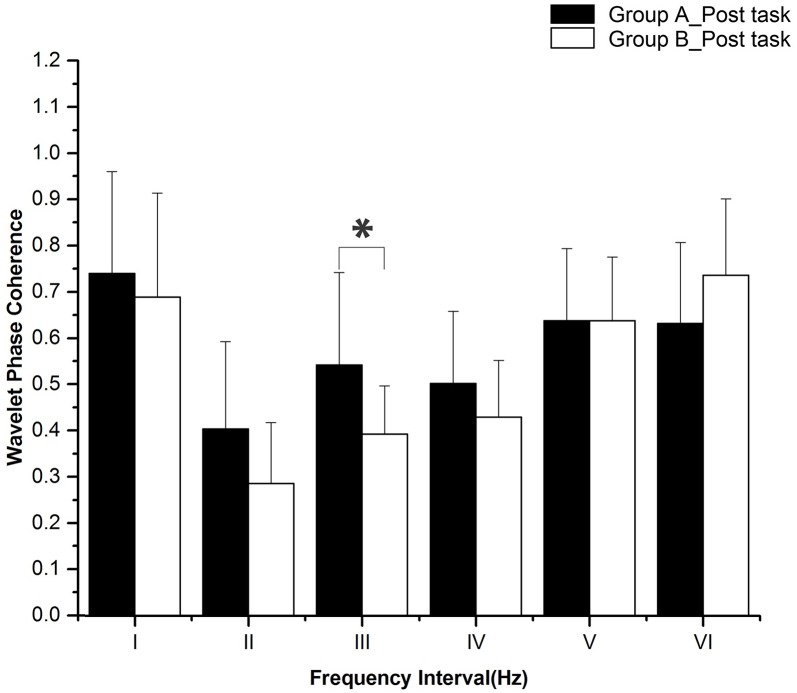
WPCO comparison in six frequency intervals in post-task recovery period between groups A and B. Significant differences are marked with *p<0.05 between the two groups. Frequency intervals: I (0.6–2 Hz); II (0.145–0.6 Hz); III (0.052–0.145 Hz); IV (0.021–0.052 Hz); V (0.0095–0.021 Hz); and VI (0.005–0.0095 Hz).

The periodic oscillations of Delta [HbO_2_] signals were identified at six frequency intervals: I: 0.6–2 Hz (cardiac activity), II: 0.145–0.6 Hz (respiratory activity), III: 0.052–0.145 Hz (myogenic activity), IV: 0.021–0.052 Hz (neurogenic activity), V: 0.0095–0.021 Hz (nitric oxide related endothelial activity) and VI: 0.005–0.0095 Hz (non-nitric oxide related endothelial activity) [32]. Sleep deprivation and task status (rest, task, and post-task recovery) did not have any significant interaction according to the two-way repeated measures ANOVA. Fig 5 shows the WPCO comparisons among subjects at rest between groups A and B. WPCO in intervals III (F = 5.955, p = 0.02) and V (F = 4.7, p = 0.037) was significantly lower in group B than in group A during the rest period. Fig 6 shows the WPCO comparison among subjects during the task period between groups A and B. WPCO in intervals III (F = 5.175, p = 0.029) and IV (F = 4.585, p = 0.039) was significantly lower in group B than in group A at task. Fig 7 illustrates WPCO comparisons among subjects at post-task recovery between groups A and B. WPCO in interval III (F = 6.125, p = 0.02) was significantly lower in group B than in group A at post-task recovery. Through Post-Hoc test after One-way ANOVA, groups A and B did not have any significant difference in WPCO among rest, task, and post-task recovery.

### Visual analogue scales

**Fig 8:**
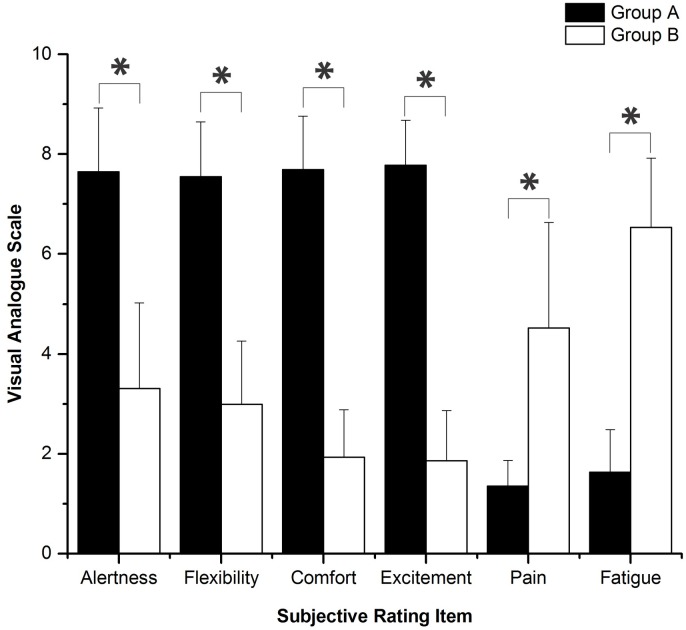
Comparison of subjective scores between two groups using VAS. Significant differences are marked with *p<0.05 between the two groups. “0” represents the worst, and “10” represents the best in alertness, flexibility, comfort, and excitement; “0” represents no pain, and “10” represents sharp pain; “0” represents no fatigue, and “10” represents severe fatigue.

The results of the comparison of several subjective scores between groups A and B are shown in Fig 8. VAS reveal that, after sleep deprivation, different degrees of decline were shown in the subjects’ alertness, flexibility, comfort, and excitement, and at the same time, a certain degree of physical pain and fatigue appeared.

### Performance evaluation

Before the alertness task, all subjects were instructed to be familiar with the task to minimize the influence of task proficiency on the test. The accuracy is calculated as follows [36]:

$$P=\frac{TP}{TP+FP}$$

In this expression, TP is the number of actual correct clicks, FP is the number of incorrect clicks, and FN is the number of incorrect misses. Moreover, F-measure is also a measure of accuracy, which considers both the precision (P) and the recall (R) of the test. The F_1_ score can be interpreted as the harmonic mean of the precision and recall, and the expression is:

$$F_{1}=\frac{2*PR}{P+R}$$

Among them, TP is the number of actual correct clicks, FP is the number of incorrect clicks, and FN is the number of incorrect misses [36].

**Fig 9:**
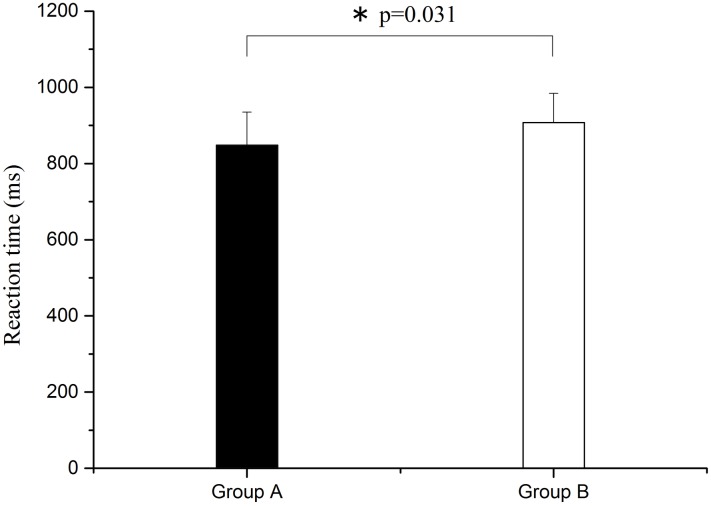
Comparison of reaction time between groups A and B. Significant difference is marked with *p<0.05 between the two groups.

As shown in Fig 9, compared with group A, reaction time was also significantly prolonged (F = 5.019, p = 0.031) after sleep deprivation.

**Fig 10:**
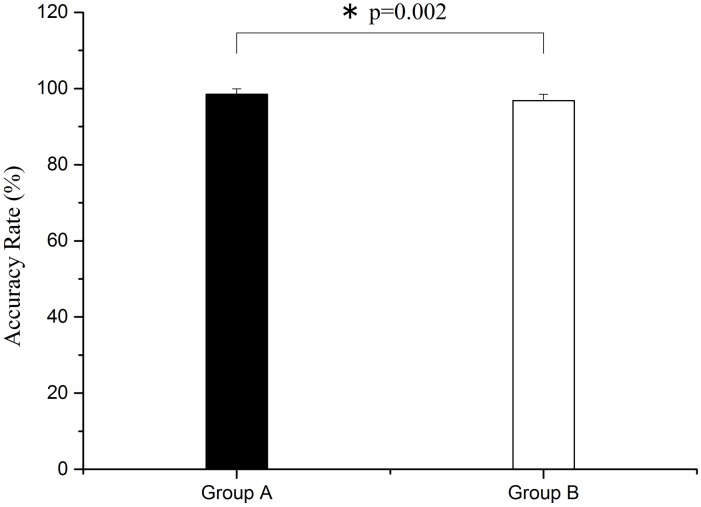
Comparison of accuracy rate between groups A and B. Significant difference is marked with *p<0.05 between the two groups.

**Fig 11:**
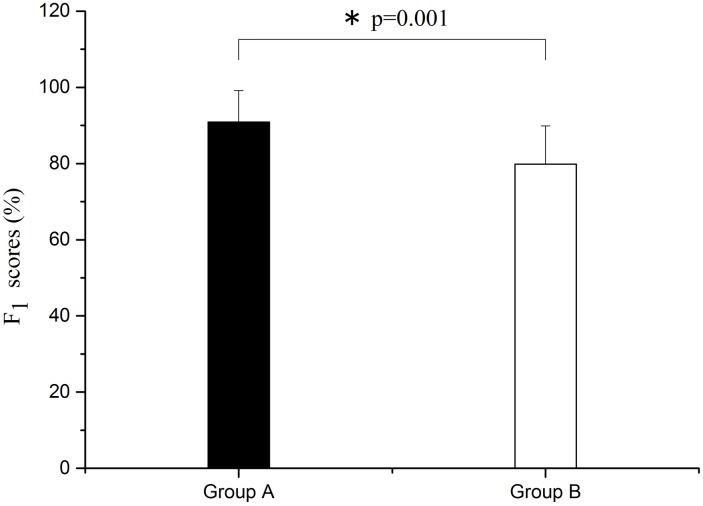
Comparison of F_1_ score between groups A and B. Significant difference is marked with *p<0.05 between the two groups.

As shown in Figs 10 and 11, through one-way ANOVA, there are significant differences both in accuracy and F_1_ score between group A and group B. Both the accuracy and the F_1_ score of group B are lower than group A (F = 11.354, p = 0.002 for accuracy rate; F = 13.708, p = 0.001 for F_1_ score).

## Discussion

The important findings of this study are as follows. (1) Phase synchronization exhibited a decline after sleep deprivation. (2) Sleep deprivation led to the reduction of alertness, flexibility, comfort, and excitement, and caused physical pain and fatigue, as assessed by VAS subjective survey. (3) After sleep deprivation, the accuracy rate and F_1_ score decreased for task execution, and the reaction time became longer. In the vigilance task, reaction time became significantly longer and accuracy rate declined considerably after sleep deprivation.

It was considerd that the cerebral NIRS originated from neurovascular coupling and systemic activity components [37]. The sympathetic nervous system, endothelial derived nitric oxide, and vascular myogenic responses could play some part in neurovascular coupling [38].

In general, the oscillations in interval I reflect the effects of cardiac activity and the interval II corresponds to respiratory activity [32]. The cerebral oscillations in interval III (0.05–0.15 Hz) originates from intrinsic myogenic activity of smooth muscle cells in resistance vessels [32] and were partly under autonomic control [39]. The interval IV is regulated by the neural control of the smooth muscle cells within the brain [32]. The oscillations in frequency intervals V and VI correspond to NO-related endothelial activity and NO-independent endothelial activity, respectively.

In this study, the interval III is consistently affected in all three states (rest, task, and post-task periods) after sleep deprivation. The sleep deprivation might affect the intravascular pressure in resistance vessels. As the vascular smooth muscles myogenic activity responses to intravascular pressure pressure in resistance vessels [32, 33], the WPCO in interval III might be consistently affected in every condition.

In this study, the resting WPCO in intervals III and V exhibited significant decrease after sleep deprivation. An imbalance in the activation existing in different brain regions was found in previous fMRI studies [4, 40] and our study is consistent with these previous studies. The prefrontal brain regions were affected by sleep deprivation. The origin of cerebral oscillation in interval III is the intrinsic myogenic activity of smooth muscle cells in resistance vessels, which is under the neural control of the cerebral circulation that is related to changes in peripheral sympathetic nerve activity [32, 39]. The reduction in WPCO values in interval III indicates that sleep deprivation leads to a reduction of phase synchronization in the left and right prefrontal lobes. This may be caused by the asymmetry of the expansion or contraction of the vessels of the left and right prefrontal lobes, which is due to sleep deprivation. This leads to the regulating effect of the intrinsic myogenic activity of the smooth muscle cells of the left and right prefrontal lobes.

The oscillations in interval V reflect the effects of NO-related endothelial activity [32, 41]. NO is a vasodilatation factor. During various circumstances, NO can be produced, released by endothelial to diastole smooth muscle cells [32]. The basis of the frequency V has been described in detail previously [33]. The decreased phase synchronization in interval V might suggest that sleep deprivation induced a de-synchronized endothelial regulation activity. Sleep deprivation has been associated with an increased incidence of metabolic disorders in humans [42]. A predominant decrease in the rate of cerebral glucose metabolism exists in the PFC after one night of sleep deprivation [42]. The metabolic activity of the frontal lobe decreases after a single night of sleep deprivation, particularly in the anterior cingulate cortex, leading to decreased performance in immediate error correction [42]. The ability to stop an initiated action can be selectively weakened by temporary deactivation of the frontal lobe [43]. These studies support our current findings.

In the task period, the WPCO in intervals III and IV in group B were significantly lower than those in group A. A functional correlation of increased activation of the left anterior PFC after sleep deprivation with short-time perception behavior was observed in the earlier study, while significant changes were not found in the right PFC activity [13]. Compared with those in the sleep-controlled condition, enhanced [HbO_2_] concentration changes are present after sleep deprivation in the left anterior PFC region of interest. A significant effect in the overall region of interest exists when the mean change in concentration in sleep-deprived condition is compared with that in sleep-controlled condition. A significant difference was also noted in each channel in the region of interest.

The oscillations in interval IV reflect the effects of neurogenic activity [32]. The two divisions of the autonomic nervous system, parasympathetic and sympathetic, which affect the heart's activities. Sympathetic vasoconstrictor and vasodilator fibers can make the vascular smooth muscle contract and relax, respectively. In addition, parasympathetic nerve fibers can make the vascular smooth muscle relax. A study shows that during vigilance testing, the significantly increased sympathetic and decreased parasympathetic extended through 24 h of sleep deprivation [44]. In a fMRI study, the right ventrolateral PFC activity was reduced at the working memory load, which indicates that degree of activation of the left and right forehead are different [13]. Hemispheric differences were also observed in a study, with the right and left PFC being more active during short memory and longer delays, respectively [45]. Studies mentioned above prove that more work load means a more asymmetric left and right prefrontal nerve activity, and that the same task experiment of cognitive sleep after sleep deprivation will increase work difficulty. Therefore, the decrease of WPCO value is caused by the asymmetry of activity in the left and right frontal nerve. This is caused by the decrease of cognitive function that results from the increase in work difficulty caused by sleep deprivation. Factors, including experimental settings, tasks performed and imaging modalities, may contribute to this discrepancy in the localized PFC activation findings between the present and previous studies [46, 47]. The increased PFC activation after sleep deprivation is also associated with neural compensation for cognitive function [48].

In post-task recovery, WPCO in interval III was significantly lower in group B than that in group A. The reduction in WPCO values in interval III reflects reduced phase synchronization in the left and right prefrontal lobes. Regulation of intrinsic myogenic activity of smooth muscle cells leads to asymmetrical blood vessel dilation or contraction capacity on both sides of the forehead, which may cause significant reduction of phase synchronization in interval III.

In this study, the WPCO shows a decrease under different conditions (rest, task, and post-task periods) after sleep deprivation. The prefrontal cortex (PFC) plays an important role in cognitive function [11]. The WPCO indicates the phase synchronization of the left and right prefrontal regions and its decline may be an indicator of the normal cognitive function [22]. In this study, VAS showed a decline in the subjects’ alertness, flexibility, comfort, and excitement, and a certain degree of physical pain and fatigue. Also, the reaction time was significantly prolonged after vigilance test, and the accuracy rate and F_1_ score declined after sleep deprivation. Combined with these results, the reduced phase synchronization between left and right prefrontal oxyhemoglobin oscillations may contribute to the decline of cognitive functions. Sleep deprivation might lead to the decline of WPCO.

Post-hoc test and the Bonferroni comparison tests were carried out during rest, task and post-task both in group A and group B. Because the sampling time of the test is 20 minutes in each interval (rest, task and post-task), and the vigilance task is relatively easy. The vigilance task did not result in fatigue and significant decrease in the cognitive function of subjects considering factors including task difficulty and sampling time. Different levels of task difficulty will lead to different levels of activity in the prefrontal lobe [8]. This is consistent with the experimental results in our previous study [49].

There is no significant difference between the two tests (for group A and group B). This is because the subjects we selected in this study are those who have good sleep quality in a month before, and this kind of acute sleep deprivation has little influence on subjects.

## Conclusions

Wavelet-based coherence analysis was used to assess the coherence between simultaneously measured left and right prefrontal Delta [HbO_2_] signals during rest, task, and post-task recovery periods in subjects with sleep deprivation. The decline in WPCO after sleep deprivation indicates reduced phase synchronization between left and right prefrontal oxyhemoglobin oscillations, which might contribute to a decline of cognitive functions.

## Supporting Information

S1 File This is the WPCO data of six frequency intervals of rest and task in group A and group B. (XLS)

S2 File This is the data of vigilance test in group A and group B, including accuracy rate, F_1_ score and reaction time. (XLS)

S3 File This is the VAS data information. (XLS)
